# Developing and validating predictive models of electronic health record tool adoption in the pre-implementation stage

**DOI:** 10.1186/s12913-026-15282-8

**Published:** 2026-08-14

**Authors:** Miguel Marino, Erin Jamieson, David Ezekiel-Herrera, Heather Holderness, Katie Fankhauser, Heather Angier, Jean O’Malley, Jennifer E. DeVoe, Nathalie Huguet

**Affiliations:** 1 Department of Family Medicine, Oregon Health & Science University, 3405 SW Perimeter Court, Portland, OR 97239-3098 USA; 2 Biostatistics Group, Oregon Health & Science University – Portland State University School of Public Health, 3181 SW Sam Jackson Park Rd, Portland, OR 97239 USA; 3 Mortenson Center in Global Engineering, University of Colorado Boulder, Boulder, CO 80309 USA; 4 Research Department, OCHIN Inc, 1881 SW Naito Pkwy, Portland, OR 97201 USA; 5 International Centre for Future Health Systems, University of New South Wales, 55 Botany Street, Randwick, Sydney, NSW 2031 Australia

**Keywords:** Machine learning, Implementation science, Cervical cancer, Community health centers

## Abstract

**Background:**

Many factors hinder adoption of implementation strategies and / or target evidence-based interventions. A gap in implementation science is the paucity of methodology for precisely assessing which medical practices will benefit most from a particular implementation strategy, how much support a medical practice might need, and if some medical practices could adopt evidence-based interventions on their own. Using machine learning, we developed and validated predictive models of the adoption of electronic health record (EHR)-related implementation strategies.

**Methods:**

This retrospective study extracted data from an EHR-based cervical cancer screening (CVC) clinical decision tool for 310 community health centers from 5/1/2017 to 6/30/2019. Machine learning models (i.e., logistic lasso regression) were used to predict adoption (any tool use in the 12 months after tool rollout vs. none) and sustained use (≥ 1 tool use in the last quarter of the 12-month study period vs. < 1 tool use in this time period). Model performance was compared using the area under the receiver operating characteristics curve (AUC) from an external, withheld clinic sample.

**Results:**

Prediction models showed good performance for adoption (AUC = 0.891, 95% CI = 0.801–0.981) and sustainability (AUC = 0.954, 95% CI = 0.889-1.000) of the CVC tool. Of the 23 EHR-derived clinic-level indicators used in the model, 18 (e.g., urbanicity) predicted adoption, and 19 (e.g., patient age) predicted sustainability of the CVC tool.

**Conclusions:**

This work provides an important step toward efficiently tailoring and deploying implementation strategies for information technology innovations.

**Supplementary Information:**

The online version contains supplementary material available at https://doi.org/10.1186/s12913-026-15282-8.

## Background

There is strong evidence that different implementation strategies (e.g., facilitation, audit and feedback, performance benchmarking, health information technology tools) can assist medical practices with adoption and maintenance of evidence-based guidelines (e.g., those for cervical cancer screening / prevention) [1–3]. Implementation strategies are systematic processes, activities, and resources which are used to enhance adoption and sustainability of an evidence-based intervention (EBI) [4]. Implementation strategies and the support that accompanies them are costly for medical practices. For instance, developing a health information technology (HIT) tool to facilitate the documentation and tracking of an EBI (e.g., cervical cancer screening) costs both the organization paying for the development of the tool, and the medical practice providing staff time to learn how to use the tool. However, many factors hinder adoption of implementation strategies and / or targeted EBIs. A gap in implementation science is the paucity of methodologies for precisely assessing which medical practices will benefit most from a particular implementation strategy, how much support a medical practice might need, and if some medical practices could adopt an EBI on their own without implementation support.

There are few methods that effectively identify the type of implementation strategies and level of support that an individual medical practice needs to implement and sustain EBIs [5]. Previous studies used pre-implementation mapping techniques to gather qualitative information but are not applicable to large scale implementation studies [6–8]. Thus, new methods are needed to predict which implementation strategy will be most effective and how much support will be needed for practices to implement targeted changes [9]. Here, we aim to determine whether electronic health record (EHR)-based clinical information could be used to predict adoption and sustained use of an EHR-based implementation strategy. For this example, we assess adoption of a clinical decision support tool (implementation strategy) developed to improve guideline concordant cervical cancer (CVC) screening and follow up care.

Decision support tools embedded within EHRs help support patients and clinical teams implement care guidelines [10–12]; such tools are an implementation strategy commonly used to improve adoption of EBIs [13]. However, often when new decision support tools are activated in EHRs, medical practices receive little support in using them. Consequently, adoption of new EHR-based or other HIT tools that could improve guideline-concordant care varies greatly, with some medical practices able to adopt them with little assistance, and others unable to do so [14, 15]. As such, predicting which medical practices are going to need extra support to adopt an EHR-based tool would be valuable. Thus, we developed and validated predictive models that estimate the likelihood of adoption and sustained use of a CVC screening clinical decision support EHR-based tool (“CVC tool”) using machine learning.

## Methods

### Data source

Using retrospective study design, we extracted EHR data for community health centers (CHCs) in 14 US states in the OCHIN (not an acronym) research network from 5/1/2017 to 6/30/2019. OCHIN is a nonprofit, practice-based research network which provides a single, patient-linked instance of the Epic^®^ EHR (each patient has a single identifier number and medical record shared across every clinic in the network) [16, 17]. We obtained OCHIN EHR data from the Accelerating Data Value Across a National Community Health Center Network (ADVANCE) clinical research network (CRN) [18]. Specifically, we extracted data from structured EHR fields from all CHC clinics live on the OCHIN EHR network for 2017 to 2019. Clinics included in this study had visits recorded during the study period (*N* = 340 CHCs), at least 100 visit records in the period prior to CVC tool implementation (pre-implementation period), at least 1 visit recorded during the study period (*N* = 311 CHCs) and a valid zip code to establish their location (*N* = 310 CHCs).

### Implementation strategy

The CVC tool was designed to improve documentation of Papanicolaou (Pap) tests, human papillomavirus (HPV) screening, and follow-up test results. The CVC tool tailors decision support suggestions for a given patient – including recommended follow-up care – based on up-to-date clinical information (e.g., latest CVC screening results). The tool also includes panel management / population tracking functions. OCHIN modified the CVC tool to create an algorithm-driven data registry that enables users to generate lists of patients due for CVC screening and follow-up care. The CVC tool can be used by various health care staff (e.g., clinic managers, providers / care teams, leadership, quality improvement staff) to identify patients due for services, assess their quality performance, and generate lists of overdue patients. The CVC tool was rolled out across OCHIN clinics in November 2018. Training consisted of clinics receiving a document indicating how to use the CVC tool.

### Outcome measures

The primary outcomes were two binary classification outcomes: adoption and sustained use of the CVC tool. Adoption was defined as any instance of tool use within 12 months of the tool roll out (November 2018-December 2019). Sustained use was defined as one or more instance of tool use in the last 4 months of the 12-month follow-up period among adopters. We chose these definitions based on known barriers of EHR tool use in the literature [e.g., lack of knowledge of the tool, lack of perceived benefit [14, 15, 19] ]. Using the tool at least once suggests that the tool was found, or its existence was known; and, using the tool repeatedly suggests the tool is seen as having some value.

### Predictor variables

We extracted 23 indicators widely available in many EHRs, including: practice size (e.g., number of visit and patients), specialty (e.g., primary care only, multidisciplinary), location (e.g., state, urban/rural classification), and patient panel characteristics (e.g., sex, race and ethnicity, health insurance). Table 1 describes each variable in detail.

**Table 1:** Description and definition of variables used in prediction models that were extracted from the electronic health record

Variables	Definition and description
**Facility characteristics**	
School based clinic	At least one department at delivery site is classified as School based at OCHIN
# eligible Mental Health departments	Eligible based on inclusion criteria: visit in pre- and post-period at delivery site
# eligible Specialty departments	Eligible based on inclusion criteria: visit in pre- and post-period at delivery site
# eligible Primary Care departments	Eligible based on inclusion criteria: visit in pre- and post-period at delivery site
Census population in zip code	Population of the delivery site’s zip code in 2010.
Urban rural class	Urban rural class of the delivery site in 2010.
Year in EHR	Mean years since the start of EHR as of 3/1/2018 among department at delivery site
Medicaid expansion state	Location of the delivery site is in a state that expanded Medicaid to ≤ 138% of the federal poverty level (FPL)
# visits	Total number of visits among departments at delivery site in pre-period
% of visit that were ambulatory	Mean % ambulatory/total visits among departments at delivery site in pre-period.
% of visit that were not ambulatory	Mean % of visits classified as non-ambulatory among departments at delivery sites in pre-period. There are three visit types: AV (ambulatory visit), OA (other ambulatory), OT (Other). Other visit types include orders only, pharmacy visit, external ed, external outpatient, external inpatient, questionnaire series submission, intake, postmortem documentation, billing visit.
% visit with healthcare provider	% total visits with physician, AP, RN, MA, or LPN provider at delivery site in pre-period
% visit with eligibility specialist	% total visits with health insurance eligibility specialist at delivery site in pre-period
**Patient panel characteristics**	
Patient median age	Mean median age of patients seen during pre-period among departments at delivery site
% female	Mean percent female patients among departments at delivery site during pre-period
% Hispanic	Mean percent Hispanic patients among departments at delivery site during pre-period
% Non-white	Mean percent non-white patients among departments at delivery site during pre-period
% Spanish speaking patient	Mean percent Spanish speaking patients among departments at delivery site during pre-period
% with multimorbidity	Mean percent patients with 2 or more chronic conditions (defined at last visit) among departments at delivery site during pre-period
% missing Federal Poverty Level (FPL)	Mean percent of patients at departments within delivery sites with no income data in pre-period
% FPL < 138%	Mean percent of patients at departments within delivery sites with last reported income in pre-period < 138 FPL.
% uninsured at visit	Mean % uninsured at ambulatory visit among departments at delivery site in pre-period.
% Medicaid at visit	Mean % Medicaid insured at ambulatory visit among departments at delivery site in pre-period.

Note: EHR, electronic health record; FPL, Federal Poverty Level

### Analysis

Our approach and reporting were informed by the TRIPOD statement for prediction models [20]. First, we randomly split our study sample into a training (i.e., development) dataset (70% of the study clinic sample), and a testing (i.e., validation) dataset (30% of the study clinic sample). Within the training dataset, 5-fold cross-validation was used to select lambda (the optimal regularization parameter). The held-out testing dataset was not used during model training or hyperparameter tuning, serving exclusively as an independent evaluation of final model performance. Of note, the random split was not explicitly stratified on the outcome since randomization (in this case with *N* = 310) typically preserves outcome prevalence balance. Descriptive analyses were conducted to report clinic characteristics, overall and by development and validation datasets.

Clinic-level inputs were assigned equal weight, regardless of the unit of measurement, by centering and scaling them [21]. Numeric inputs were scaled to have a mean of 0 and a standard deviation of 0.5. The observed distributions in the training dataset were independently applied to the testing dataset.

To build our prediction models, we utilized logistic lasso regression [22], which is a regularized regression method providing a better approach for prediction and variable selection than other well-known methods such as stepwise forward / backward selections. In short, regularization methods (like the logistic lasso regression) impose a constraint on the size of the coefficients and force some regression estimates to be exactly zero, thus achieving variable selection while shrinking the rest of the coefficients to address overfitting of the prediction model [23]. LASSO was selected because it simultaneously performs regularization and variable selection in moderate-sized samples (such as ours), it produces interpretable coefficients which is important for implementation scientists, and yields results that are easier to integrate into EHR systems, increasing potential for real-world adoption. We used stratified 5-fold cross-validation and specified the ensemble to minimize rank loss while maximizing the area under the receiver operating characteristic curve (AUC). The performance of these models was compared using the AUC from an externally withheld validation clinic sample. All analyses were completed using R version 4.0.3. This study was approved by the Institutional Review Board at our academic health center.

## Results

A total of 310 clinics representing 526 clinical departments met study criteria. Characteristics of clinics and the patients they serve are reported in Table 2. The majority of clinics were urban (92.3%), in a state that expanded Medicaid to ≤ 138% of the federal poverty level (FPL,) and had been using their EHR for an average of 4 years. The average number of visits in the baseline period was 87,421. The demographic characteristics of patients seen in study clinics during the baseline period included: mean age of 32 years (SD = 14), 57% female, 27% Hispanic, 33% had 2 or more chronic conditions, 55% had Medicaid coverage, 20% were uninsured, and 63% had incomes of < 138% FPL. Overall, the distributions of patient characteristics were similar between the development and validation datasets. Appendix Table 1 describes the characteristics of the clinics and patients they serve by tool use groups.

**Table 2:** Clinic characteristics overall and by testing and development data sets

Variables	Total sampleMean (SD) or N (col %	TestingMean (SD) or N (col %)	DevelopmentMean (SD) or N (col %)
# of clinics	310	93	217
School based clinic	87 (28.1)	25 (26.9)	62 (28.6)
Population in zip code	26710 (18922.09)	26030 (16041.78)	27002 (20057.71)
Urban clinic	286 (92.3)	85 (91.4)	201 (92.6)
Years in EHR	4.56 (2.63)	4.84 (2.75)	4.44 (2.58)
Medicaid expansion state	279 (90.0)	84 (90.3)	195 (89.9)
# eligible Mental Health departments	0.32 (0.66)	0.27 (0.63)	0.34 (0.68)
# eligible Specialty departments	0.15 (0.62)	0.15 (0.46)	0.15 (0.68)
# eligible Primary Care departments	1.02 (0.69)	0.98 (0.55)	1.04 (0.74)
Patient median age	32 (14.06)	33 (14.68)	31 (13.76)
% female	56.52 (12.91)	55.55 (14.54)	56.93 (12.15)
% Hispanic	26.75 (25.28)	26.23 (25.08)	26.98 (25.42)
% Non-white	28.69 (25.95)	27.85 (25.09)	29.06 (26.36)
% Spanish speaking patients	16.00 (18.64)	16.27 (19.76)	15.88 (18.18)
% with multimorbidity	33.45 (20.82)	36.16 (23.07)	32.29 (19.73)
% missing FPL	24.88 (27.67)	23.05 (26.18)	25.67 (28.30)
% FPL <138%	63.19 (27.38)	65.07 (26.00)	62.39 (27.98)
# visits	87422 (269976.00)	62292 (171640.31)	98192 (302212.79)
% of visit that were ambulatory	58.14 (20.99)	59.10 (21.22)	57.73 (20.93)
% of visit that were not ambulatory	0.88 (2.26)	0.66 (1.23)	0.97 (2.58)
% uninsured at visit	19.74 (21.35)	19.48 (19.67)	19.85 (22.07)
% Medicaid at visit	55.13 (21.86)	55.91 (20.93)	54.80 (22.29)
% visit with healthcare provider	73.87 (28.62)	69.17 (30.46)	75.88 (27.63)
% visit with eligibility specialist	0.10 (0.55)	0.10 (0.50)	0.09 (0.58)

Note: EHR, electronic health record; FPL, Federal Poverty Level

**Fig. 1:**
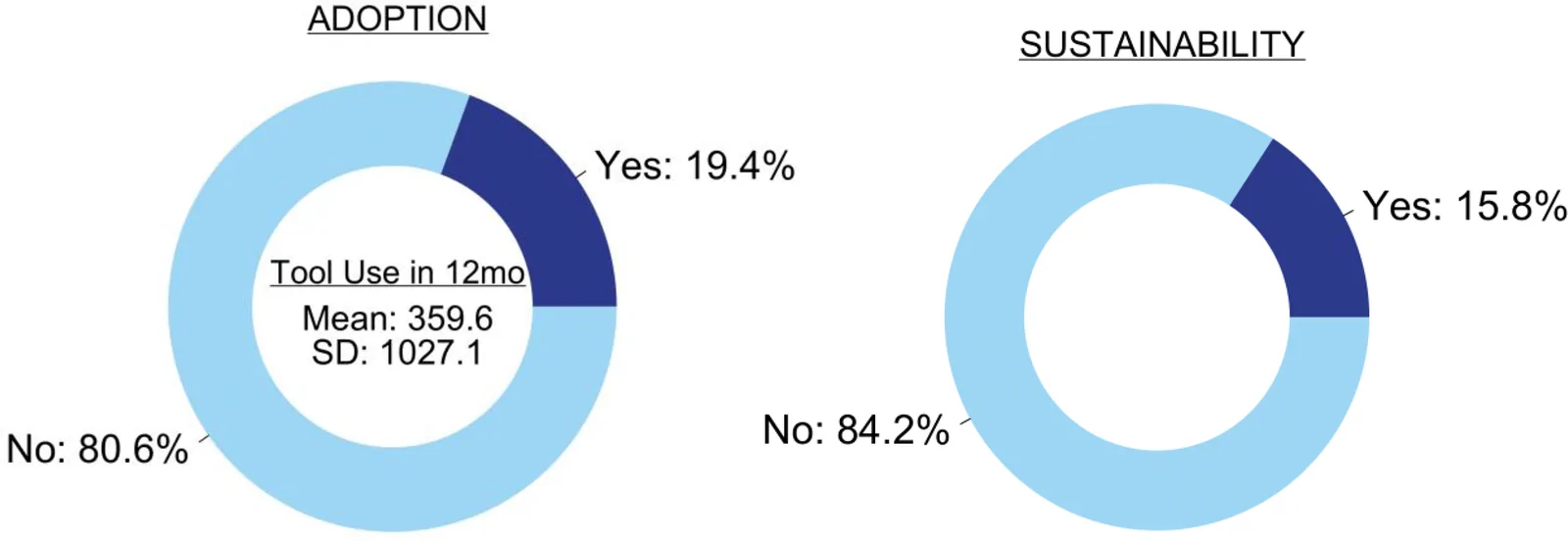
Prevalence of adoption and sustainability of the cervical cancer screening tool. Descriptive statistics of tool use over the 12-month follow-up period among tool users

As denoted in Fig. 1, of the 310 clinics, 19.4% met the criteria for adoption of the CVC tool in the 12 months after rollout, and 15.8% met the criteria for sustainability. Among clinics that adopted the CVC tool, the average number of times the tool was used over the follow-up period was 360 (SD = 1,027).

Using the validation dataset to assess predictive performance (*N* = 93), AUCs for adoption (AUC = 0.891, 95% CI = 0.801–0.981) and sustainability (AUC = 0.954, 95% CI = 0.889-1.000) showed outstanding predictive performance (Table 3). Of the 23 EHR-derived inputs that went into the CVC tool prediction model, 18 were important for prediction of adoption and 19 for prediction of sustainability. Of note, the variables that showed lower odds of both adoption and sustainability of the CVC tool included: being a school-based clinic, having a higher population in the clinic’s zip code, having higher number of specialty departments, having higher percentage of Spanish-speaking patients, having higher percentage of ambulatory visits, and having a higher percentage of visits with eligibility specialists relative to their counterparts. Alternatively, several variables showed higher odds of adoption and sustainability including a clinic being in an urban setting, more years with an EHR, being in a state that expanded Medicaid coverage, having a higher percentage of female and non-white patients, having a higher percentage of patients with multimorbidity, having more visits overall, having a higher percentage of non-ambulatory visits and uninsured visits, and having a higher percentage of visits with a healthcare provider relative to their counterparts (Table 3). A list of all coefficients for the LASSO prediction models is reported in Appendix Table 2.

**Table 3:** Predictive model performance in withheld validation data set for the cervical cancer screening tool adoption and sustainability outcomes and description of retained variables and their relation to each outcome

	CVC Tool		
	Adoption	Sustainability	
**Model Performance**			
AUC (95% CI)	0.891 (0.801, 0.981)	0.954 (0.889, 1.000)	
**Variables**			
School based Clinic	Lower OR	Lower OR	
Population in Zip code	Lower OR	Lower OR	
Urban clinic	Higher OR	Higher OR	
Years in EHR	Higher OR	Higher OR	
Medicaid expansion state	Higher OR	Higher OR	
# eligible Mental Health departments	--	--	
# eligible Specialty departments	Lower OR	Lower OR	
# eligible Primary Care departments	Higher OR	--	
Patient median age	--	Lower OR	
% female	Higher OR	Higher OR	
% Hispanic	--	--	
% Non-white	Higher OR	Higher OR	
% Spanish speaking patients	Lower OR	Lower OR	
% with multimorbidity	Higher OR	Higher OR	
% missing FPL	--	Higher OR	
% FPL < 138%	--	Higher OR	
# visits	Higher OR	Higher OR	
% of visit that were ambulatory	Lower OR	Lower OR	
% of visit that were not ambulatory	Higher OR	Higher OR	
% uninsured at visit	Higher OR	Higher OR	
% Medicaid at visit	Lower OR	--	
% visit with healthcare provider	Higher OR	Higher OR	
% visit with eligibility specialist	Lower OR	Lower OR	

Note: -- indicates variables not selected to be important for prediction of the outcomeCVC: cervical cancer screening, OR: odds ratio, EHR, electronic health record; FPL, Federal Poverty Level

## Discussion

This study demonstrates how prediction models can help clinics prepare for the implementation of new tools, and to precisely estimate which clinics are likely to adopt an EHR tool before implementation begins. This study makes a significant contribution to the field of implementation science by creating a novel, low-budget method for employing “*precision implementation.*” There has long been a recognition that “one size does not fit all” when it comes to implementing new strategies (e.g., EHR tools); however, the inability to predict the support needed and likelihood of successful implementation has greatly hampered implementation efforts. This study helps to solve this problem. By using widely available and easily accessible EHR data, implementation scientists and practitioners can use these *precision implementation* methods to better plan for tool implementation, including identifying the clinics that will need extra support to adopt and sustain an EHR-based tool.

In this study, adoption of the CVC EHR tool was low, clearly indicating the need for additional support to increase uptake. As in previous studies, uptake of EHR-related support tools varies widely across clinics and depends on the type of tool [24–26]. Yet, EHR-related tools have been shown to improve guideline concordant care [27–30]. Using a machine learning approach, we were able to predict, with good accuracy, the clinics who adopted and sustained use of the HIT tool (i.e., CVC tool). Although prediction algorithms are imperfect and cannot replace working directly with clinics, this novel approach may be useful for resource planning and sampling decisions (i.e., which clinics are unlikely to implement a strategy). In addition, *precision implementation* could help scientists augment or avoid complex, time intensive, and resource consuming clinic assessments, (i.e., Change Process Capability Questionnaire) [31].

We used 23 clinic-level indicators, and although they are typically available, some may be more challenging to obtain (e.g., percent of patients with multimorbidity) than others depending on the clinic’s capacity to extract EHR data. Our results showed several indicators were predictive of both adoption and sustainability including types of clinic (i.e., school based, multidisciplinary), years with an EHR, percent of patients who were Spanish speaking, and percent of ambulatory or specialist visits. Implementation scientists could target these indicators to evaluate the likelihood of adoption of an EHR tool.

A strength of this study was the large number and heterogeneity of the clinics, which allowed for sufficient variations across the 23 indicators to estimate odds of adoption and sustainability given a variety of combinations of predictors. This study has limitations. There are no establish thresholds to define adoption or sustainability. Using alternate definitions may lead to different modeling performance and predictors. The predictors of the CVC tool may not perform as well for predicting adoption of another clinical tool. In addition, the sample of clinics was restricted to CHCs, and our results may not be applicable in non-CHC primary care clinics. Future studies are needed to test this model on other EHR tools and in different settings.

In conclusion, we developed and validated novel prediction models and the use of available EHR data to determine the likelihood of adoption and sustained use of an EHR-related tool in a multistate network of CHCs. These prediction models are proof of concept that such models could be applied in future trials to predict which implementation strategies might benefit a clinic with certain characteristics, and that using this novel ‘*precision implementation*’ method can precisely tailor and adapt implementation strategies (and implementation support) to meet the needs of specific clinical practice settings.

## Supplementary Information

Below is the link to the electronic supplementary material.

Supplementary Material 1

## Data Availability

Raw data underlying this article were generated from multiple health systems across institutions in the OCHIN Network; restrictions apply to the availability and re-release of data under organizational agreements.

## Abbreviations

EBI: Evidence based intervention
HIT: Health information technology
EHR: Electronic health records
CHC: Community health centers
ADVANCE: Accelerating Data Value Across a National Community Health Center Network
CVC: Cervical cancer screening
TRIPOD: Transparent Reporting of a multivariable prediction model for Individual Prognosis Or Diagnosis
AUC: Area under the receiver operating characteristic curve
FPL: Federal poverty level

## References

[1] Grimshaw JM, Thomas RE, MacLennan G, Fraser C, Ramsay CR, Vale L, et al. Effectiveness and efficiency of guideline dissemination and implementation strategies. Health Technol Assess. 2004;8(6):iii–iv. 10.3310/hta8060 14960256

[2] Prior M, Guerin M, Grimmer-Somers K. The effectiveness of clinical guideline implementation strategies–a synthesis of systematic review findings. J Eval Clin Pract. 2008;14(5):888–97. 10.1111/j.1365-2753.2008.01014.x 19018923

[3] Graham R, Mancher M, Miller Wolman D, Greenfield S, Steinberg E, editors. Clinical practice guidelines we can trust. Washington (DC); 2011. 24983061

[4] Proctor EK, Powell BJ, McMillen JC. Implementation strategies: recommendations for specifying and reporting. Implement Sci. 2013;8:139. 10.1186/1748-5908-8-139 PMC3882890 24289295

[5] Powell BJ, Beidas RS, Lewis CC, Aarons GA, McMillen JC, Proctor EK, et al. Methods to Improve the Selection and Tailoring of Implementation Strategies. J Behav Health Serv Res. 2017;44(2):177–94. 10.1007/s11414-015-9475-6 PMC4761530 26289563

[6] Lewis CC, Scott K, Marriott BR. A methodology for generating a tailored implementation blueprint: an exemplar from a youth residential setting. Implement Sci. 2018;13(1):68. 10.1186/s13012-018-0761-6 PMC5956960 29769096

[7] Lewis CC, Fischer S, Weiner BJ, Stanick C, Kim M, Martinez RG. Outcomes for implementation science: an enhanced systematic review of instruments using evidence-based rating criteria. Implement Sci. 2015;10:155. 10.1186/s13012-015-0342-x PMC4634818 26537706

[8] Fernandez ME, Ten Hoor GA, van Lieshout S, Rodriguez SA, Beidas RS, Parcel G, et al. Implementation Mapping: Using Intervention Mapping to Develop Implementation Strategies. Front Public Health. 2019;7:158. 10.3389/fpubh.2019.00158 PMC6592155 31275915

[9] Lewis CC, Weiner BJ, Stanick C, Fischer SM. Advancing implementation science through measure development and evaluation: a study protocol. Implement Sci. 2015;10:102. 10.1186/s13012-015-0287-0 PMC4511441 26197880

[10] Bright TJ, Wong A, Dhurjati R, Bristow E, Bastian L, Coeytaux RR, et al. Effect of clinical decision-support systems: a systematic review. Ann Intern Med. 2012;157(1):29–43. 10.7326/0003-4819-157-1-201207030-00450 22751758

[11] Ravikumar KE, MacLaughlin KL, Scheitel MR, Kessler M, Wagholikar KB, Liu H, et al. Improving the Accuracy of a Clinical Decision Support System for Cervical Cancer Screening and Surveillance. Appl Clin Inf. 2018;9(1):62–71. 10.1055/s-0037-1617451 PMC5801884 29365341

[12] Sequist TD, Zaslavsky AM, Marshall R, Fletcher RH, Ayanian JZ. Patient and physician reminders to promote colorectal cancer screening: a randomized controlled trial. Arch Intern Med. 2009;169(4):364–71. 10.1001/archinternmed.2008.564 PMC2683730 19237720

[13] Powell BJ, Waltz TJ, Chinman MJ, Damschroder LJ, Smith JL, Matthieu MM, et al. A refined compilation of implementation strategies: results from the Expert Recommendations for Implementing Change (ERIC) project. Implement Sci. 2015;10:21. 10.1186/s13012-015-0209-1 PMC4328074 25889199

[14] Jetelina KK, Woodson TT, Gunn R, Muller B, Clark KD, DeVoe JE, et al. Evaluation of an Electronic Health Record (EHR) Tool for Integrated Behavioral Health in Primary Care. J Am Board Fam Med. 2018;31(5):712–23. 10.3122/jabfm.2018.05.180041 PMC6261664 30201667

[15] Ramsey A, Lord S, Torrey J, Marsch L, Lardiere M. Paving the Way to Successful Implementation: Identifying Key Barriers to Use of Technology-Based Therapeutic Tools for Behavioral Health Care. J Behav Health Serv Res. 2016;43(1):54–70. 10.1007/s11414-014-9436-5 PMC4362852 25192755

[16] Devoe JE, Sears A. The OCHIN community information network: bringing together community health centers, information technology, and data to support a patient-centered medical village. J Am Board Fam Med. 2013;26(3):271–8. 10.3122/jabfm.2013.03.120234 PMC3883432 23657695

[17] Devoe JE, Gold R, Spofford M, Chauvie S, Muench J, Turner A, et al. Developing a network of community health centers with a common electronic health record: description of the Safety Net West Practice-based Research Network (SNW-PBRN). J Am Board Fam Med. 2011;24(5):597–604. 10.3122/jabfm.2011.05.110052 PMC3525325 21900444

[18] DeVoe JE, Gold R, Cottrell E, Bauer V, Brickman A, Puro J, et al. The ADVANCE network: accelerating data value across a national community health center network. J Am Med Inf Assoc. 2014;21(4):591–5. 10.1136/amiajnl-2014-002744 PMC4078289 24821740

[19] Huguet N, Ezekiel-Herrera D, Gunn R, Pierce A, O’Malley J, Jones M, et al. Uptake of a Cervical Cancer Clinical Decision Support Tool: A Mixed-Methods Study. Appl Clin Inf. 2023;14(3):594–9. 10.1055/s-0043-1769913 PMC10411153 37532232

[20] Collins GS, Reitsma JB, Altman DG, Moons KG. Transparent reporting of a multivariable prediction model for individual prognosis or diagnosis (TRIPOD): the TRIPOD statement. BMJ. 2015;350:g7594. 10.1136/bmj.g7594 25569120

[21] Gelman A. Scaling regression inputs by dividing by two standard deviations. Stat Med. 2008;27(15):2865–73. 10.1002/sim.3107 17960576

[22] Tibshirani R. Regression Shrinkage and Selection via the Lasso. J Roy Stat Soc. 1996;58(1):267–88.

[23] McNeish DM. Using Lasso for Predictor Selection and to Assuage Overfitting: A Method Long Overlooked in Behavioral Sciences. Multivar Behav Res. 2015;50(5):471–84. 10.1080/00273171.2015.1036965 26610247

[24] Liberati EG, Ruggiero F, Galuppo L, Gorli M, Gonzalez-Lorenzo M, Maraldi M, et al. What hinders the uptake of computerized decision support systems in hospitals? A qualitative study and framework for implementation. Implement Sci. 2017;12(1):113. 10.1186/s13012-017-0644-2 PMC5602839 28915822

[25] Khairat S, Marc D, Crosby W, Al Sanousi A. Reasons For Physicians Not Adopting Clinical Decision Support Systems: Critical Analysis. JMIR Med Inf. 2018;6(2):e24. 10.2196/medinform.8912 PMC5932331 29669706

[26] Gold R, Bunce A, Davis JV, Nelson JC, Cowburn S, Oakley J, et al. I didn’t know you could do that: A Pilot Assessment of EHR Optimization Training. ACI open. 2021;5(1):e27–35. 10.1055/s-0041-1731005 PMC8691746 34938954

[27] Magrath M, Yang E, Ahn C, Mayorga CA, Gopal P, Murphy CC, et al. Impact of a Clinical Decision Support System on Guideline Adherence of Surveillance Recommendations for Colonoscopy After Polypectomy. J Natl Compr Canc Netw. 2018;16(11):1321–8. 10.6004/jnccn.2018.7050 PMC7210781 30442733

[28] Mahmoud AS, Alkhenizan A, Shafiq M, Alsoghayer S. The impact of the implementation of a clinical decision support system on the quality of healthcare services in a primary care setting. J Family Med Prim Care. 2020;9(12):6078–84. 10.4103/jfmpc.jfmpc_1728_20 PMC7928113 33681044

[29] Atlas SJ, Tosteson ANA, Wright A, Orav EJ, Burdick TE, Zhao W, et al. A Multilevel Primary Care Intervention to Improve Follow-Up of Overdue Abnormal Cancer Screening Test Results: A Cluster Randomized Clinical Trial. JAMA. 2023;330(14):1348–58. 10.1001/jama.2023.18755 PMC10565610 37815566

[30] Nierengarten MB. Improving follow-up of abnormal cancer screening results: Basic interventions and reminders in the electronic health record can help increase the benefits of timely follow-up for patients. Cancer. 2024;130(3):332. 10.1002/cncr.35191 38300739

[31] Balasubramanian BA, Marino M, Cohen DJ, Ward RL, Preston A, Springer RJ, et al. Use of Quality Improvement Strategies Among Small to Medium-Size US Primary Care Practices. Ann Fam Med. 2018;16(Suppl 1):S35–43. 10.1370/afm.2172 PMC5891312 29632224

