# Supplementary material for: Developing and validating predictive models of electronic health record tool adoption in the pre-implementation stage
Source: BMC Health Serv Res. 2026 Aug 14;26:1583. doi: 10.1186/s12913-026-15282-8 (PMC13760908; doi:10.1186/s12913-026-15282-8)
Supplement: Supplementary file 1 [file 12913_2026_15282_MOESM1_ESM.docx]

| **Appendix Table 1**. Characteristics of clinics by cervical cancer tool use adoption and sustainability. | | | | | |  |  |  |
| --- | --- | --- | --- | --- | --- | --- | --- | --- |
|  | **Adoption** | | **Sustainability** | | | | | |
| **Variables** | Tool Use  Mean (SD) or N (col %) | No Tool Use Mean (SD) or N (col %) | | Sustained Use Mean (SD) or N (col %) | Not Sustained Use Mean (SD) or N (col %) | |  |  |
| ***# of clinics*** | 60 | 250 | | 49 | 261 | |  |  |
| School based clinic | 2 (3.3) | 85 (34.0) | | 2 ( 4.1) | 85 (32.6) | |  |  |
| Population in zip code | 21549 (14701) | 27948 (19625) | | 21979 (14787) | 27598 (19497) | |  |  |
| Urban clinic | 58 (96.7) | 228 (91.2) | | 48 (98.0) | 238 (91.2) | |  |  |
| Years in EHR | 5.0 (2.7) | 4.5 (2.6) | | 5.1 (2.9) | 4.5 (2.6) | |  |  |
| Medicaid expansion state | 54 (90.0) | 225 (90.0) | | 46 (93.9) | 233 (89.3) | |  |  |
| # eligible Mental Health departments | 0.43 (1.00) | 0.29 (0.55) | | 0.45 (1.08) | 0.30 (0.55) | |  |  |
| # eligible Specialty departments | 0.42 (1.21) | 0.08 (0.33) | | 0.51 (1.32) | 0.08 (0.32) | |  |  |
| # eligible Primary Care departments | 1.42 (1.11) | 0.92 (0.50) | | 1.47 (1.19) | 0.93 (0.50) | |  |  |
| Patient median age | 36 (10) | 30 (15) | | 36 (9) | 31 (15) | |  |  |
| % female | 60.3 (10.4) | 55.6 (13.3) | | 61.0 (8.0) | 55.7 (13.5) | |  |  |
| % Hispanic | 19.7 (20.1) | 28.5 (26.1) | | 20.8 (20.1) | 28.0 (26.0) | |  |  |
| % Non-white | 33.9 (30.5) | 27.4 (24.6) | | 31.1 (28.4) | 28.3 (25.5) | |  |  |
| % Spanish speaking patient | 12.1 (14.9) | 16.9 (19.3) | | 12.7 (14.6) | 16.6 (19.3) | |  |  |
| % with multimorbidity | 38.8 (12.9) | 32.2 (22.1) | | 39.8 (12.2) | 32.3 (21. 9) | |  |  |
| % missing FPL | 30.8(26.5) | 23.5 (27.8) | | 34.5 (27.0) | 23.1 (27.5) | |  |  |
| % FPL <138% | 55.8 (27.5) | 65.0 (27.1) | | 52.8 (27.2) | 65.2(27.0) | |  |  |
| # visits | 384651 (517817) | 16086 (22781) | | 456524 (548080) | 18126 (25901) | |  |  |
| % of visit that were ambulatory | 45.5 (15.0) | 61.2 (21.1) | | 43.9 (14.0) | 60.8 (21.0) | |  |  |
| % of visit that were not ambulatory | 1.00 (1.30) | 0.85 (2.43) | | 1.09 (1.38) | 0.8 (2.4) | |  |  |
| % uninsured at visit | 18.4 (24.3) | 20.1 (20.6) | | 15.6 (21.0) | 20.5 (21.5) | |  |  |
| % Medicaid at visit | 49.1 (21.2) | 56.6 (21.8) | | 50.7 (19.0) | 56.0 (22.3) | |  |  |
| % visit with healthcare provider | 87.1 (10.3) | 70.7 (30.6) | | 87.2 (10.2) | 71.4 (30.3) | |  |  |
| % visit with eligibility specialist | 0.02 (0.14) | 0.11 (0.61) | | 0.02 (0.15) | 0.11 (0.60) | |  |  |
| Note: EHR, electronic health record; FPL, Federal Poverty Level | | | | | | | |  |

| **Appendix Table 2**. Coefficients of LASSO prediction models for the cervical cancer tool adoption and sustainability outcomes | | | |
| --- | --- | --- | --- |
| **Variables** | **Adoption** | **Sustainability** |  |
| School based Clinic | -2.608 | -2.906 |  |
| Population in Zip code | -0.538 | -1.219 |  |
| Urban clinic | 1.044 | 1.101 |  |
| Years in EHR | 0.383 | 0.330 |  |
| Medicaid expansion state | 1.596 | 0.670 |  |
| # eligible mental health departments | -- | -- |  |
| # eligible Specialty departments | -3.093 | -1.654 |  |
| # eligible Primary Care departments | 0.083 | -- |  |
| Patient median age | -- | -1.038 |  |
| % female | 0.478 | 0.629 |  |
| % Hispanic | -- | -- |  |
| % Non-white | 1.244 | 0.093 |  |
| % Spanish speaking patients | -2.649 | -1.962 |  |
| % with multimorbidity | 0.26 | 1.057 |  |
| % missing FPL | -- | 1.687 |  |
| % FPL <138% | -- | 1.946 |  |
| # visits | 25.726 | 19.968 |  |
| % of visit that were ambulatory | -0.598 | -1.405 |  |
| % of visit that were not ambulatory | 0.497 | 0.829 |  |
| % uninsured at visit | 2.301 | 2.735 |  |
| % Medicaid at visit | -0.874 | -- |  |
| % visit with healthcare provider | 2.001 | 3.220 |  |
| % visit with eligibility specialist | -0.610 | -0.572 |  |
| Intercept | -1.461 | -2.746 |  |
| Note: EHR, electronic health record; FPL, Federal Poverty Level | | |  |
